# Integrating Primary and Secondary Care to Enhance Chronic Disease Management: A Scoping Review

**DOI:** 10.5334/ijic.5508

**Published:** 2021-02-09

**Authors:** Sara Murtagh, Geoff McCombe, John Broughan, Áine Carroll, Mary Casey, Áine Harrold, Thomas Dennehy, Ronan Fawsitt, Walter Cullen

**Affiliations:** 1 School of Medicine, University College Dublin, Ireland; 2 School of Nursing Midwifery and Health Systems, University College Dublin, Ireland; 3 Ireland East Hospital Group, Dublin, Ireland

**Keywords:** integrated care, primary care, secondary care, scoping review, chronic disease

## Abstract

**Background:**

In Ireland, as in many healthcare systems, health policy has committed to delivering an integrated model of care to address the increasing burden of chronic disease. Integrated care is an approach to healthcare systems delivery that aims to minimise fragmentation of patient services and improve care continuity. To this end, how best to integrate primary and secondary care is a challenge. This paper aims to undertake a scoping review of empirical work on the integration of primary and secondary care in relation to chronic disease management.

**Methods:**

A search was conducted of ‘PubMed’, ‘Cochrane Library’ and ‘Google Scholar’ for papers published between 2009–2019 using Arksey and O’Malley’s framework for conducing scoping reviews.

**Results:**

Twenty-two studies were included. These reported research from a wide range of healthcare systems (most commonly UK, Australia, the Netherlands), adopted a range of methodologies (most commonly randomised/non-randomised controlled trials, case studies, qualitative studies) and among patients with a range of chronic conditions (most commonly diabetes, COPD, Parkinson’s disease). No studies reported on interventions to address the needs of whole populations. Interventions to enhance integration included multidisciplinary teams, education of healthcare professionals, and e-health interventions. Among the effectiveness measures reported were improved disease specific outcomes, and cost effectiveness.

**Conclusion:**

With healthcare systems increasingly recognising that integrated approaches to patient care can enhance chronic disease management, considerable literature now informs how this can be done. However, most of the research published has focussed on specific diseases and their clinical outcomes. Future research should focus on how such approaches may improve health outcomes for populations as a whole.

## Introduction

Chronic diseases especially diabetes, cardiovascular disease, chronic obstructive pulmonary disease (COPD), and cancer are the leading causes of mortality globally and are major contributors to healthcare costs [1]. To complicate matters, these conditions are often multifaceted in nature, and affected patients frequently have complex care needs, many of which cannot be adequately met by overly fragmented primary and secondary care services [2]. Further, as the population of older adults grows, the need for effective chronic disease prevention and management programmes also grows, and this need can be met by integrated forms of health and social service provision [2]. ‘Integrated care’ is an approach to healthcare delivery which aims to remedy the fragmentation of patient services and improve continuity of care. The patient, his/her family and his/her community are placed at the centre of care [3]. It should be noted there is no universally agreed understanding of ‘integration’ as it is considered an umbrella term with about 175 different definitions and concepts [4]. With primary care’s main function as a point of first contact, continuous, comprehensive, and coordinated care, it has a central role in coordinating and integrating care, particularly in the patient centred model [5].

While numerous definitions of integrated care exist in the literature, a common theme throughout is the ‘patient-centred’ nature of integrated care. According to the World Health Organisation, integrated service delivery is…

“…the organization and management of health services so that people get the care they need, when they need it, in ways that are user-friendly, achieve the desired results and provide value for money” [6, p. 1].

Such patient-centred versions of care can exist in different structures; the form of integrated care that this literature review is concerned with is the integration between primary and secondary care. The term ‘collaborative care’ is often used in conjunction with similar models of care, however some commentators have suggested that this term should not be used interchangeably with ‘integrated care’, as the two hold separate meanings [7].

An integrated approach to care delivery for patients with chronic disease can enhance health outcomes [8]. Integrating primary and secondary healthcare has the potential to enhance communication and access to care therefore promoting health and improving patient satisfaction and participation. It may also have the potential to reduce unnecessary spending and increase cost effectiveness within healthcare systems [2,9]. The World Health Organisation recommends ensuring continuity of care through efficient and cost-effective systems of referral and communication between primary and secondary services with GPs acting at the centre of multi-professional teams from the health, social and other sectors [2]. Successful integrated care requires the ongoing involvement of patients and family in care planning, implementation, and oversight [3]. This self-management empowers patients and ensures the delivery of patient-centred individualised care. Developing such integrated systems to address the growing burden of chronic disease is a priority. The treatment and management of patients with chronic illnesses is responsible for 80% of GP visits, 40% of hospital admission, and 75% of hospital bed days [1].

In Ireland, the challenge of chronic disease prevention and management is currently being addressed by numerous directives, most notably those set out by the ‘Sláintecare’ programme [10]. This policy was published by the Irish Government in 2017, and it promotes a unified and long-term vision for health policy, healthcare, and social care services in Ireland [10]. With respect to the management of chronic disease, Sláintecare advocates moving away from the current hospital-focussed model and providing care closer to home for patients. The programme asserts that when chronic disease conditions are effectively managed in the community setting, clinical outcomes may improve, thus delivering “better value-for-money and maintain strong focus on health promotion and public health with the aim of preventing chronic disease from overwhelming the health service in the future” [10].

Taking an integrated care approach to address the needs of patients should also be associated with improved outcomes. However, trials have produced mixed results with some yielding higher hospital admissions and costs reference source. This suggest some of the presumed benefits may not be possible in practice and more research is needed into the effective implementation of integrated care [11]. This scoping review aims to identify the extant literature on integrated approaches to care for patients with chronic disease and to identify priority areas to focus on in future research and implementation. In line with the directives of Sláintecare, the study will focus on the role of integration in the management of chronic disease patients in community-based primary care settings.

## Methods

A search of the extant literature to identify the integrated approaches to care for patients with chronic disease and identify priority areas to focus on in future research and implementation was undertaken in the form of a scoping review. The scoping review method was chosen because the topic under investigation has previously yielded mixed research findings, thus creating uncertainty concerning the benefits of integrated care for chronic disease management [11]. Such uncertainty restricts the ability to formulate and test well defined hypotheses using experimental research methods, and so it was decided that a more inductive investigation approach was required. Scoping review methods are well suited to tasks of this nature as they aim to map the literature on a research area and provide an opportunity to identify key concepts, knowledge gaps and evidence [12]. The scoping review framework used in this study comprises an iterative six-stage process, as developed by Arksey and O’Malley [13] with later recommendations by Levac et al. [14]. These stages were:

### Stage 1: Identifying the research question

The aim of this review focused on establishing priority areas for future research to enhance integration of primary and secondary care and access to healthcare based on current knowledge and identifiable gaps. Primary care was defined as comprehensive, accessible health services provided by practitioners who address many personal health care needs, develop sustained partnership with patients and practice within a family and community setting [15]. This definition was chosen as it recognizes the importance of three perspectives for primary care: the patient and family, the community, and the integrated delivery system. While integrated care does not have one universally agreed upon definition, for the purpose of this review the WHO definition of integrated care as “the organization and management of health services so that people get the care they need, when they need it, in ways that are user-friendly, achieve the desired results and provide value for money” was chosen.

From this understanding, the following two research question were formed:

1. What is known about the successful implementation of integrated primary and secondary care;
2. What areas of research should be prioritised to inform the process of successful integration and the best possible outcomes in the Irish context?

### Stage 2: Identifying relevant studies

The comprehensive three-step search strategy recommended by the Joanna Briggs Institute (JBI) systematic reviews [16] was utilized to identify both published and unpublished (‘grey’) literature. The first step was an initial search of relevant databases such as PubMed, followed by an analysis of the text words contained in the title and abstract, and of the index terms used to describe articles. A second search using all identified keywords and index terms was then undertaken across all included databases. Thirdly, the reference list of all identified reports and articles were manually searched for additional relevant studies. Only studies published in English were considered for inclusion. The search strategy was limited to studies published in the last ten years and conducted in EU, Canada, or Australia due to the similar (i.e. two-tiered) nature of the healthcare systems of these countries.

Electronic databases searched included: PubMed, Google Scholar and Cochrane library. Electronically available peer-reviewed journals were hand-searched. Additionally, the International Journal of Integrated Care was identified as a key publication and was hand-searched separately to identify relevant studies that were not identified through the databases search. Citations were managed using the bibliographic software manager ‘EndNote x9’. Ultimately the search terms used were the MeSH term “delivery of healthcare” and “integrated” or “integration” or “integrating” as well as the related terms “primary-secondary care interface”, “collaborative care” and “shared care”.

### Stage 3: Study selection

**Figure 1:**
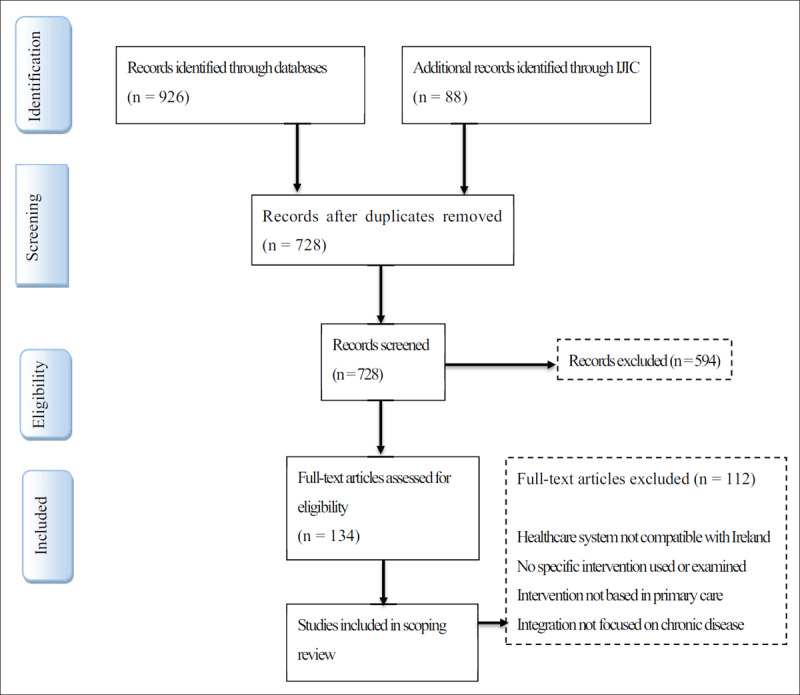
Flowchart illustrating the paper selection process.

The selection process consisted of two levels of screening: (1) a title and abstract review and (2) a full-text review. To ensure that only relevant studies were included, screening was conducted by two reviewers, a medical student (SM) and a post-doctoral researcher (GM). These reviewers also managed any conflicts arising with regards to the study selection process. The PRISMA flow diagram outlining the study selection results is outlined in ***Figure 1***. Consistent with scoping review methodology, this study was broad in its inclusion of different types of literature [13,14,17], and an assessment of methodological quality was not performed. Both peer-reviewed and grey literature were searched, with no methodological requirement for study inclusion. This facilitated the inclusion of an array of literature, which included quantitative, qualitative, and mixed-method studies, as well as systematic reviews and meta-analyses. Protocols were excluded.

The initial search identified 936 studies with a further 88 added from the International Journal of Integrated Care, total (n = 1014). Two hundred and eighty-six duplicates were removed leaving 728 studies for further examination. The selection process involved an initial screening of the title and abstract and then a full-text review of remaining articles. Studies were selected using the following inclusion criteria:

- Published in the date range 2009–2019
- English language
- Studies undertaken in countries with two-tier healthcare systems (e.g. Ireland, EU, Canada, or Australia)
- Investigating specific interventions
- Integration in the primary care setting

Due to the large volume of articles identified a further inclusion criterion was added limiting results to those relating to interventions focussing on improving chronic disease care. The PRISMA flow diagram as illustrated in Fig. 1 outlines the results of the literature search.

### Stage 4: Charting the data

Data from included articles were organized to facilitate comparison and thematic analysis (***Table 1***). The following data were extracted from articles:

**Table 1:** Summary of key findings from studies identified


AUTHOR, YEAR	COUNTRY	STUDY POPULATION	TITLE	STUDY DESIGN	INTERVENTION	MAIN FINDINGS

Hollingworth et al, 2017 [18]	Australia	Type 2 diabetes	Impact of a general practitioner-led integrated model of care on the cost of potentially preventable diabetes-related hospitalisations.	Non-randomised controlled trial	Special interest GP led multidisciplinary team with an endocrinologist and diabetes educator – Beacon model of care	– Estimated savings from potentially preventable hospitalisations = €79.1 million – Reduction in cost and occurrence of preventable hospital admissions – Works on a small scale, local approach.

Eggers et al, 2018 [19]	Germany	Parkinson’s disease	Patient-centred integrated healthcare improves quality of life in Parkinson’s disease patients: a randomized controlled trial	Randomised controlled trial	Community-based multidisciplinary team with PD specialist, nurse and general neurologist	– Significant improvement in QoL (PDQ-39), motor and non-motor symptoms – Cost benefit & maintenance inconclusive – Multidisciplinary expertise and nurse most innovative aspects – Improved patient empowerment in terms of disease acceptance or coping

Kruis et al, 2010 [20]	Netherlands	COPD	Sustained effects of integrated COPD management on health status and exercise capacity in primary care patients	Non-randomised controlled trial	Multidisciplinary team with two physiotherapists, respiratory nurse, physician assistant, dietician, pharmacist, supervising primary care physician and logistics manager	– Improvement in health status and exercise tolerance – Better results as a primary care base than other studies – Suggestion cost may improve from early intervention – Need for prolonged intervention. – Improved feelings of self-efficacy, control over one’s own disease state.

Kruis et al, 2014 [21]	Netherlands	COPD	Effectiveness of integrated disease management for primary care chronic obstructive pulmonary disease patients: results of cluster randomised trial.	Randomised controlled trial	General practitioners, practice nurses, and specialised physiotherapists in the intervention group received a two-day training course on incorporating integrated disease management in practice	– No significant difference in QoL, outcomes, self-management – Activity levels improved – Improvements in follow-up structure – Freedom for each team to decide the integration plan and priorities that worked best for them. Less intense but more realistic

Fortin et al, 2016 [22]	Canada	Multiple chronic diseases	Integration of chronic disease prevention and management services into primary care: a pragmatic randomized controlled trial (PR1MaC).	Randomised controlled trial	Patient centred self-management support and health education with interprofessional collaboration. Started with a preliminary clinical evaluation by a trained nurse and development of intervention plan based on the patient’s objective discipline involved.	– Modest yet beneficial effects of the intervention – Improvement in positive and active engagement in life, social integration and support – Much room for improvement, intervention too short

Zhang et al, 2015 [23]	Australia	Type 2 diabetes	Impact of an integrated model of care on potentially preventable hospitalizations for people with Type 2 diabetes mellitus	Open controlled trial	Care provided by multidisciplinary team of an endocrinologist; two/three advanced trained general practitioners, a diabetes educator, and a podiatrist, with additional allied health available on referral	– Intervention group half as likely to be hospitalised for potentially preventable db related incident – Shorter hospital stay: no evidence that the severity of conditions differed – Suggests substantial improvements in healthcare utilisation costs as well as patient outcomes

Boland et al, 2015 [24]	Netherlands	COPD	Cost-effectiveness of integrated COPD care: the RECODE cluster randomised trial	Randomised controlled trial	Multidisciplinary team using personally developed plan to redesign and integrate care process following a 2-day training course. Incorporated ICT application with patient and provider portal to measure and report process and outcomes.	– Not cost effective, higher cost and no significant difference in effects – Relatively low intensity of pragmatic intervention -teams not required to implement all elements learned during courses – Limited room for improvement due to the relatively high standard of care

Ferrone et al, 2019 [25]	Canada	COPD	The impact of integrated disease management in high-risk COPD patients in primary care.	Randomised controlled trial	Integrated disease management, self-management, and structured follow-up intervention: case management, self-management education, and skills training. Team care model, shared decision making Supported by electronic point-of-service system	– QoL improved – Significantly fewer sever exacerbations in IDM patients – Less hospitalisations (not statistically significant) – Improved COPD knowledge – Better lung function, FEV1

Busetto et al, 2015 [26]	Netherlands	Type 2 diabetes	Implementation of integrated care for diabetes mellitus type 2 by two Dutch care groups: a case study.	Embedded single case study, data collected through semi-structured interviews	Care groups, bundled payments, patient involvement, health professional’s cooperation and task substitution, evidence-based care protocols and a shared clinical information system Electronic administration and exchange of data.	– Quality of care increased effective, efficient, accessible, patient-centred, equitable and safe health care. – Insufficient integration between patient data bases, decreased earnings for some health professionals – Improved communication and cooperation – Need more attention on patient and community involvement.

Hernandez et al, 2015 [27]	Spain, Norway, Greece	Multiple chronic diseases	Integrated care services: lessons learned from the deployment of the NEXES project.	Randomised controlled trial	An Integrated Care Service with the aim of achieving target objectives aligned with a comprehensive treatment plan based on their health condition and social circumstances.	– Wellness and Rehabilitation service did not show significant positive effects in two of the sites – Positive outcomes in prevention of hospitalizations in high risk patients in Spain and Greece – The level of evidence on effectiveness raised in NEXES was uneven for the different services – Demonstrates effectiveness of IT-supported care services, with potential for cost-containment and complementariness of the deployment of integrated care services

Van der Marck et al, 2013 [28]	Netherlands	Parkinson’s disease	Integrated multidisciplinary care in Parkinson’s disease: a non-randomised, controlled trial (IMPACT)	Non-randomised controlled trial	Individually tailored assessment by a multidisciplinary team to create a comprehensive treatment plan and subsequent implementation of the plan within a network of specifically trained allied health professionals and follow up by the same team nurse.	– Small improvements in favour of the intervention. – Significant improvements in anxiety and depression, activities of daily living, non-motor symptoms and perceived general health – Quality-of-care scores were better but overall satisfaction was unchanged – Economic evaluation showed the average costs were similar in both groups

Waibel et al, 2015 [29]	Spain	COPD	The performance of integrated health care networks in continuity of care: a qualitative multiple case study of COPD patients.	Qualitative multiple case study	Case studies with joint management care which showed similarities in level of internal production of services but differences regarding the integration depth and inter-organisational relationship. The networks introduced different types of care coordination mechanisms ranged from an implemented single mechanism (shared electronic medical records, shared clinical guidelines, COPD patient registers, etc.) to a combination of mechanisms in a comprehensive programme	– Clear distribution of COPD care responsibilities – GPs followed the instructions received from the specialists and incorporated it into treatment plan But pulmonologists recorded disregarding recommendations from the other care level – High accessibility to care during exacerbations but long waiting times to non-urgent care – Continuity of care facilitated via computer – Established trusting patient–physician relationship over time

Russell et al, 2013 [30]	Australia	Type 2 diabetes	Model of care for the management of complex Type 2 diabetes managed in the community by primary care physicians with specialist support: an open controlled trial.	Open controlled trial	Care in the community by GPs with advanced skills, supported by an endocrinologist. Screening assessment by a diabetes nurse educator. Support from a dietician, psychologist and podiatrist as needed Developed a patient-specific management plan.	– Consistently lower HbA1c after 6 months – Blood pressure and total cholesterol initially significantly reduced but equivalent to usual care group at 12 months – The intervention group had higher proportions of patients’ clinical outcome targets – Self-efficacy improved – Glycaemic control improved with HbA1c consistently lower – 2.7 times as many patients seen per session – Intervention model delivered at approximately one fifth of the cost per visit accessibility

Russell et al, 2019 [31]	Australia	Type 2 Diabetes	Clinical outcomes of an integrated primary-secondary model of care for individuals with complex type 2 diabetes: a non-inferiority randomised controlled trial.	Randomised controlled trial	Beacon model of care using a multidisciplinary team including two GPs with special interests, an endocrinologist and a DNE in a community general practice. GPs took online advanced diabetes care course and attended a 1-day workshop	– More doctors’ visits and DNE appointments – Difficulty obtaining a better average HbA1c – No differences in BP or lipids – No difference to their QoL but higher satisfaction with care scores, although this difference was small – Better self-management support – Participants found clinicians collaborative, which improved engagement and motivation. – Higher number of doctor and DNE visits may reflect improved patient access and real-time follow-up

Hanan et al, 2014 [32]	Ireland	Cancer	Delivering care to oncology patients in the community: an innovative integrated approach	Robust evaluation of pilot	Community oncology nurse education programme to provide continuous professional development enabling nurses to develop and enhance their knowledge, skill and competence. The training is both theoretical and skills based. The national implementation groups comprises senior clinical oncology nurses, managers and educationalists.	– Considered successful by both the community and hospital staff – Improved QoL – Dramatic decrease in hospital attendances for clinical procedures that are now performed in the community – Increase in community nurses’ confidence and competency in providing a safe service in the patient’s home. – Communication between the hospital and community staff was strengthened by the training programme – Increased sense of autonomy

Oude et al, 2015 [33]	Netherlands	Diabetes	Effects of Government Supervision on Quality of Integrated Diabetes Care: a Cluster Randomized Controlled Trial	Cluster randomised controlled trial	Care groups of multiple health care providers, general practitioners and practice nurses provide diabetes care. Practice nurses perform check-ups. Bundled payment means paying a single fee for all medical services care	– Structures and processes of care did not improve more than usual, neither did health outcomes – Could not demonstrate an effect of the supervision program on quality of care in care groups – Explanations are no effect of the supervision program, control group improved too, limiting contrast or the effect of the program was not captured in this study design.

Browne et al, 2016 [34]	Australia	Type 2 diabetes	Building the evidence for integrated care for type 2 diabetes: a pilot study.	Pilot evaluation	IDEAS: an integrated, multidisciplinary, community-based health service Multidisciplinary team including an endocrinologist and registrar working with a diabetes nurse educator, podiatrist and community health nurse.	– No effect on diabetes-specific distress scores – Diabetes-specific self-efficacy did not change significantly over time as a result of receiving care – Participants perceived the quality of diabetes care as significantly better with a person-centred focus – No significant difference in HbA1c, trend in favour of IDEAS but did not reach significance

Yu et al, 2017 [35]	UK	Type 2 diabetes	Population-level impact of diabetes integrated care on commissioner payments for inpatient care among people with type 2 diabetes in Cambridgeshire: a postintervention cohort follow-up study.	Post intervention study	Community diabetes service with increased specialist nursing, dietetic, podiatry and medical support to primary care and patients, while linking into other diabetes specialist services	– Lower individual median inpatient payment – Successfully implemented with positive patient experience, improved practice nurse clinical confidence and early reports of clinical benefit – Failed to progress to a truly integrated services, potentially related failure to implement integrated information management

Simmons et al, 2015 [36]	UK	Diabetes	Hospitalisation among patients with diabetes associated with a Diabetes Integrated Care Initiative: a mixed methods case study.	Mixed methods case study	Three-component model involving GP, hospital and community with the intermediate service led by community-based diabetes specialist nurses Increased access to patient diabetes education, greater within-practice diabetes specialist support for primary care, increased linkage with hospital diabetes specialists	– Increase patients diagnosed with diabetes was comparable, prevalence increased during this time – No improvement in diabetes QoF targets – No significant reductions, and no differences compared with the other two areas, in hospitalisation rates for diabetes – A feeling that intervention increased barriers to direct access to hospital services rather than facilitating due to new additional layers to their care – Better level of personalised care than they had previously – Negatively affected by a lack of functioning information-sharing systems – Failure to implement information management systems probably led to communication and integration difficulties

Burridge et al, 2015 [37]	Australia	Diabetes	The work of local healthcare innovation: a qualitative study of GP-led integrated diabetes care in primary health care	Qualitative study	Community care led by GPs with advanced skills, supported by an endocrinologist and a diabetes nurse educator. Support from a dietician, psychologist and podiatrist as needed	– Intervention challenges professional norms and involves changes to traditional delivery models and renegotiation of professional roles – Success dependent on the trust of all involved and the credibility of clinicians – Findings imply a deeper, potentially resistant mindset – Highlighted the influence of macro institutional processes on micro-level professional identities and work practices

Hepworth et al, 2013 [38]	Australia	Type 2 diabetes	‘Working with the team’: an exploratory study of improved type 2 diabetes management in a new model of integrated primary/secondary care.	Interviews	A multidisciplinary team of an endocrinologist, advanced-skilled GPs, a diabetes educator and a podiatrist	– Participant reported easy to use, and more convenient than travelling to major hospitals for routine procedures – Diabetes advice readily accessible and participants felt comfortable requesting advice – Staff identified as “very supportive”, “very helpful” and providing care on a “one-to-one basis” – Improved patient understanding of diabetes and how to manage it, empowered to be active in their own health

Burridge et al, 2017 [39]	Australia	Type 2 diabetes	A qualitative follow-up study of diabetes patients’ appraisal of an integrated diabetes service in primary care	Interviews	Multidisciplinary clinics led by skilled GPs with special interest in diabetes and includes assessment screening and blood glucose stabilisation Model informed by elements of the Chronic Care Model, redesigning the delivery system and improving patients’ self-management skills – to improve care efficiency and effectiveness	– Increased convenience for patients with shorter wait times – Clinicians had the flexibility to tailor specialised information to the patients’ specific circumstances – Patients positioned as partners in care which increased engagement and self-care – Fluidity of boundaries might work best with meeting patients’ healthcare needs

- First author, year published
- Study Location
- Study population
- Name of study
- Study design
- Intervention used
- Major findings

### Stage 5: Collating, summarizing, and reporting results

Data was collated to provide an overview of the breadth of the literature and to aid with presentation of findings. Following this the major themes of the literature were identified. As this is a scoping review, no assessment of quality of evidence was performed.

### Stage 6: Consultation

In line with recommendations by Levac et al. [14], studies were also included and excluded according to the outcomes of consultation with experts in the field of healthcare and healthcare research.

## Results

### Search results

Initial screening identified 728 studies in total. 594 studies were excluded based on title and abstract which revealed studies that were not based in the included countries, were not published in the included date range, were not based in primary care, or studies such as reviews or study protocols. The remaining 134 were read and a further 112 were excluded due to full text not available or not available in English, not examining chronic disease interventions and those revealed to also not be based in the included countries or a primary care setting. A total of 22 studies from 2009–2019 were included in the final data synthesis.

To assist in the organisation of the scoping review, the literature was structured into six areas: study design, intervention studied, clinical outcomes, cost effectiveness, electronic integration, and patient/healthcare providers’ experiences.

### Study design

The final 22 studies included in this review were both quantitative and qualitative. There were eight randomised controlled trials, three non-randomised and two open controlled trials. There was also one embedded single case study with data collected through semi-structured interviews, one qualitative multiple case study, two pilot evaluations and one postintervention study. The remaining four studies were qualitative studies.

### Population

Of the included studies, 20 examined populations with a specific chronic illness and two studied multiple chronic conditions [22,27]. The most common disease type was diabetes with 12 of the 22 studies examining populations with the diabetes [18,2326^30^,^31^,^33^,^34^,^35^,^36^,^37^,^38^,^39^]. Five studies examined patients with COPD [20,2124^25^,^29^], two examined patients with Parkinson’s disease [19,20,21,22,23,24,25,26,27,28] and one study examined patients with cancer [32].

### Intervention studied

A key element across almost all the studies is the incorporation of a multidisciplinary team in the integrated care intervention used. The structure of these teams usually varied depending on their purpose. That is, the teams consisted of professionals suited to managing the health conditions which they sought to address. Many teams had either a GP leading [18,30,39] or a nurse coordinating care [28]. For most part, studies documenting multidisciplinary teams also demonstrated that regular meetings and/or remote contact between team members working in primary and secondary care services can be effective in terms of ensuring swift transitions between care levels, thus enhancing patient safety and continuity of care. An educational component was included in some studies [22,25,32] particularly those with a study population of patients with diabetes [35,38]. Interventions ranged from short term randomised controlled trials to long term larger scale pilot programmes. The time involved ranged from a few months to multiple years. It is suggested that some of the expected outcomes of various studies may not have been achieved due to a time frame that was insufficient for allowing intervention improvements to come in to effect [33,39].

### Clinical outcomes

Results varied in terms of clinical outcomes. Some studies showed distinct improvements [20,28] with less severe exacerbations [25], fewer potentially preventable hospitalisations [23] and better outcomes in disease specific markers [30]. On the other hand, some studies had little to no significant effect on clinical outcomes or only small changes in a few [33]. In some instances, this is attributed to a lack of uniformity among individual approaches within an intervention group [24] or a lack of patient specific focus [21].

### Cost-effectiveness

Numerous studies supported the idea that integrated healthcare is a more cost-effective healthcare delivery system [18,35]. This is in part due to better use of resources or reduced hospital admissions [23]. However, some studies which predicted a greater cost effectiveness found that there was little or no significant difference between the intervention groups and the control groups [19,24].

### Electronic integration

One element that can be seen across the examples of the integrated healthcare studies was the integration and support of electronic health records [24,25]. This facilitated communication between the different healthcare professionals, primary and secondary care levels, and in the multidisciplinary intervention setting. This allowed all of a patient’s information to be compiled in one area and maintained the patient centred philosophy of an integrated care intervention. Studies that failed to properly integrate IT systems attribute some of the negative results or unplanned losses to this lack of electronic support [26,35,36]. Further, it was noted that ineffective electronic integration processes can significantly hinder integration between primary and secondary care, with patients highlighting that such systems can lead to the patients becoming ‘carriers’ of information between care levels.

### Patient/professional experience

Across many of the studies it was found that patients perceived quality of life to be improved with the implementation of an integrated primary and secondary care system [19,25,28,32]. In studies where patients’ clinical outcomes or cost effectiveness failed to produce positive results, many still showed a positively reported patient experience and support for the continued use of an integrated care model [21,34]. Studies showed an improvement in patients’ self-efficacy, autonomy and confidence in understanding and managing their own illnesses [30,32,38]. The healthcare professionals involved in the integrated care teams also showed strong communication and positive interrelationships while working as part of a multidisciplinary team [27,31,32]. Although there were some difficulties with the transition from well-defined roles to the less clearly structured integrated system [37].

## Discussion

### Key findings

This study aimed to map the literature concerning the integration of primary and secondary care to enhance chronic disease management. In line with the directives of Irish healthcare policy (i.e. Sláintecare), the study focused on reviewing articles that examined such integration from a primary care perspective. Our findings indicate that integrating primary and secondary care can enhance clinical outcomes and is cost-effective for patients with specific chronic illnesses (i.e. diabetes, COPD, Parkinson’s disease). Among those interventions we identified, were multidisciplinary teams, education of healthcare professionals, and e-health interventions. There appears to be a paucity of research examining whole populations with chronic illnesses, specific integrated care interventions, and literature from Ireland.

### How this relates to other literature

In 2011, the World Medical Association recommended that healthcare systems ‘advocate for integration of chronic disease prevention and control strategies in government-wide policies’ and ‘focus on providing primary care training opportunities that highlight the integrative and continuity elements of the primary care specialties including family medicine’ [40]. Since then, various models have been established which aim to integrate secondary healthcare services with primary, community-based care for the treatment and management of chronic diseases. Such models include interventions such as primary care staff education and the implementation of multidisciplinary teams across healthcare settings. This review aimed to establish priority areas for future research to enhance integration of primary and secondary care and access to healthcare based on current knowledge and identifiable gaps. No intervention model provided uniform results across all populations and settings. The results of this scoping review suggest it is important to consider the specific population as well as the economic, social, and healthcare context within which the intervention is implemented when considering the reasons for its success.

The lack of conclusive results for the cost effectiveness of integrated care suggests that policy-makers and healthcare professionals cannot depend on significant savings in the shorter term of the implementation of most interventions, such as multidisciplinary teams, staff education and electronic data exchange. However, long-term savings were found in some studies and this finding may have been replicated if the interventions implemented in other studies were continued for a longer duration. Also, it is worth noting that caution should always be exercised when interpreting cost-effectiveness results, as these figures are often subject to great variance in reliability and reproducibility. On a positive note, across almost all the studies, patient outcomes or satisfaction were improved in one way or another whether it be from better clinical outcomes, quality of life or interpretation of quality of care.

Another point to note is that although there is quite a high volume of literature relating to the integration of care, there is a much smaller number of practical applications or investigations of specific interventions. The number of studies examining the investigation of a specific integrated care intervention for the treatment of chronic diseases was relatively low especially considering the importance placed upon integrated care as the method for alleviating the growing burden of chronic diseases. In particular, a notable limitation of the reviewed studies was that bar discussion of the electronic integration approaches, they often failed to provide close examination of communication strategies between primary and secondary care services (e.g. regarding referral procedures, service agreements, e-consults), as well as these strategies effects on patient and service level outcomes. The absence of such examination limits the studies’ ability to inform future research and practice, as effective communications is widely considered to be a fundamental component of successful integrated care models [2]. Furthermore, only two of the studies included in this scoping review considered multiple chronic diseases suggesting a gap in research relating to interventions that can successfully cater to multiple different chronic diseases simultaneously in the primary care setting. For example, such interventions could include the integration of chronic disease prevention and management services into primary care settings or educational interventions for primary care practitioners.

### Implications for research, education, and practice

Although there is an abundance of research examining various integrated care interventions, research specific to the integration of primary and secondary care in the treatment of chronic illness is less common. There was a lack of conclusive evidence as to the success of particular intervention types, or elements of interventions (e.g. communication strategies), with results varying across outcomes, settings and population types. More research is required to determine best practice in the integration of primary and secondary care for the treatment of chronic illness. It is important that common elements of interventions, such as the use of a multidisciplinary care team and education of healthcare professionals, be identified.

The care models identified were often specific to a particular disease, suggesting more research is required to examine how best to effectively treat multiple chronic diseases in an integrated care setting. Only two studies examined multiple chronic conditions, of which one found modest yet beneficial improvements as a result of the intervention [22], and one failed to find significant positive effects [27]. This finding poses the question as to whether the use of particular models of care mitigate integration of care. More research examining treatment of conditions that are multi-morbid in nature, in an integrated setting is warranted.

Furthermore, there was also only one study [21] conducted in Ireland which may suggest a priority area for future research. The findings of this review, which highlight the importance of the social and economic context in the success of interventions, indicate that although a particular intervention is found to be successful in one healthcare setting, these findings may not be replicated in the context of the healthcare setting of another country. More research on integrated models of care for chronic illness in the Irish healthcare setting are warranted to inform the implementation of Ireland’s Sláintecare programme, thus formulating the most successful variation of integrated care interventions in the Irish healthcare setting.

### Methodological considerations

The adoption of a scoping review methodology benefitted this study as the method allowed us to map the literature concerning integrated care for chronic disease conditions. This mapping of the literature resulted in us being able to provide a clear overview of what is an area of research characterised by mixed, and thus puzzling, research findings. The use of Arksey and O’Malley’s scoping review framework was also beneficial, as the framework ensured that our research development, study selection, and data interpretation processes were conducted using a widely accepted and therefore suitably rigorous approach. However, there were some limitations to our review, which should be considered when interpreting the findings. Firstly, the scoping review methodology itself gives rise to some limitations. While we aimed to be comprehensive in our approach, there is a possibility that not all publications relevant to the subject area were identified by the search strategy. In addition, scoping reviews do not include an assessment of study quality as the focus is on covering the range of work rather than limiting the work to studies that meet particular methodological criteria. Secondly, only articles published in English were considered for inclusion in our review, which could have resulted in the exclusion of relevant literature published in other languages.

## Conclusion

This review sought to establish priority areas for future research to enhance integration of primary and secondary care, and access to healthcare based on current knowledge and identifiable gaps. A broad array of outcomes was identified across 22 reviewed articles, which were categorized under the wide-ranging themes of clinical outcomes, cost-effectiveness, electronic integration and professional/patient experience. The benefits and need for integrated care are well discussed but there lacks a particular model which has been proven to improve all outcomes in the Irish healthcare context. This demonstrates the importance of taking into account specific populations and intervention strategies, as well as the economic and social context, when examining the effectiveness of particular models of integrated care.

This review established priority areas for future research, particularly the integrated management of multiple chronic conditions, and integrated models of primary and secondary care in the Irish healthcare context. While considerable literature has examined the integration of primary and secondary care to enhance chronic disease management, the vast majority of studies focused on one particular disease type. Given the multi-morbid nature of many chronic diseases, and the growing burden of such diseases on society, further research examining new models of care to enhance multiple diseases is a priority. Furthermore, future research is warranted in the Irish healthcare setting in order to establish which models of integrated care demonstrate the most success in this setting.
